# Genomic Landscape of 6597 Hong Kong HBOC Patients: Implications for Beyond-BRCA Multi-Gene Panel Testing and Cancer Surveillance

**DOI:** 10.3390/ijms27177572

**Published:** 2026-08-24

**Authors:** Ava Kwong, Cecilia Y. S. Ho, Sze Keong Tey, Chun Hang Au, Edmond S. K. Ma

**Affiliations:** 1 Department of Surgery, The University of Hong Kong, Hong Kong SAR, China; 2 Breast Surgery Centre and Cancer Genetics Center, Hong Kong Sanatorium & Hospital, Hong Kong SAR, China; 3 Hong Kong Hereditary Breast Cancer Family Registry, Hong Kong SAR, China; 4 Division of Molecular Pathology, Department of Pathology, Hong Kong Sanatorium & Hospital, Hong Kong SAR, China; 5 Research Department, Hong Kong Sanatorium & Hospital, Hong Kong SAR, China

**Keywords:** germline, Chinese, hereditary breast–ovarian cancer, multi-gene pane, beyond *BRCA*

## Abstract

Breast cancer remains highly prevalent, where the lifetime risk before age 75 is one in 13. However, known genetic factors were only identified in 14.7% of cases in our Hong Kong Hereditary Breast Cancer Family Registry. Our current local policy for genetic testing does not cover the detection of beyond *BRCA1/2*. Here we highlight the clinical value of extending testing to beyond *BRCA* susceptibility genes for improved prevention, diagnosis, and management. We recruited 6597 hereditary breast and ovarian cancer (HBOC) patients from our registry based on family history and clinical criteria. Germline mutations were identified by multi-gene sequencing analysis using next-generation sequencing (NGS). Clinical–pathological characteristics of *BRCA* and beyond *BRCA* carriers were compared and the real-world management and surveillance services adopted in Hong Kong were highlighted. In this multi-gene hereditary cancer cohort, germline mutations were identified in 10.9% of cases for *BRCA1/2* and 3.5% for beyond *BRCA* susceptibility genes. These beyond *BRCA* mutations constitute a considerable proportion of actionable hereditary risk. Notably, *PALB2* emerged as the most prevalent non-*BRCA* gene, followed by *TP53*, *ATM*, and *BARD1*. New cancers or recurrences were detected during their surveillance; the overall pick up rates were 8.3% (*PALB2*), 29.4% (*TP53*), 33.3% (*PTEN*) and 5.6% (*BARD1*). This study provides the first comprehensive characterization of the beyond *BRCA* germline landscape in a Hong Kong hereditary cancer cohort and highlights the current need for implementing multi-gene sequencing analysis and surveillance services for HBOC patients, establishing the predominant non-*BRCA* drivers and providing a robust empirical basis for expanding public genetic screening frameworks.

## 1. Introduction

Breast cancer is the most common cancer worldwide and has contributed to a significant impact on global cancer-related deaths [1]. According to 2023 data from the Hong Kong Cancer Registry, breast cancer has been the second most frequently diagnosed cancer among the population and ranked first in females with 5585 new cases a year [2]. The lifetime risk before age 75 is one in 13 with a median age of diagnosis at age 60 [2]. Approximately 5–10% of breast cancer cases are hereditary breast cancer, developed in carriers of mutations in breast cancer susceptibility genes [3]. Data from our Hong Kong Hereditary Breast Cancer Family Registry (Table 1) reveals that known hereditary factors have been identified in 14.7% of our HBOC breast and/or ovarian cancer patients selected for genetic testing due to high-risk clinical factors. It has been estimated that heritable factors can contribute to up to 27% of breast cancer cases [4]. Breast cancer is among the most common cancers with genetic susceptibility, with *BRCA1* and *BRCA2* regarded as the most frequently identified genes. The germline pathogenicity rate for *BRCA1* and *BRCA2* consistently hovers around 9.4% to 10.6% of HBOC breast and/or ovarian cancer patients. Multi-gene panel testing has led to the detection of pathogenic/likely pathogenic (P/LP) variants in cancer susceptibility genes in patients with breast–ovarian cancer spectrum. Besides *BRCA1/2*, P/LP variants in high- and moderate-penetrance genes for breast–ovarian cancer, including *TP53*, *CDH1*, *PALB2*, *STK11*, *PTEN*, *CHEK2*, *ATM*, *BARD1*, *BRIP1*, *RAD51C* and *RAD51D*, were identified after the advent of next-generation sequencing (NGS)-based testing. Many studies have shown that non-*BRCA* cancer susceptibility genes contribute to increased breast and other cancer risk [5,6].

Germline *BRCA1* and *BRCA2* testing has been integrated into many national healthcare systems worldwide and in Hong Kong. The Hong Kong Hospital Authority, the main body of public health services in Hong Kong, has started offering germline *BRCA1* and *BRCA2* testing to all newly diagnosed ovarian cancer and invasive breast cancer patients since October 2021 and July 2025 respectively, enabling the identification of high-risk individuals who may benefit from the knowledge about their cancer predisposition in terms of oncological prevention, diagnostics, and therapeutic options. The clinical practice guidelines from the U.S. National Comprehensive Cancer Network (NCCN) recommended genetic testing other than *BRCA1* and *BRCA2* in 2020. However, the testing policies for non-*BRCA* genes still vary among countries and such genetic testing is still not yet being subsidized by the public medical sectors in Hong Kong, but only sponsored by a charitable organization (the Hong Kong Hereditary Breast Cancer Family Registry). Therefore, we aim to identify the prevalence of hereditary cancer in Hong Kong patients with HBOC spectrum, and the contribution of non-*BRCA* breast cancer susceptibility genes detected by multi-gene panel testing in order to highlight the recognition, diagnosis, and treatment and provide prevention recommendations to patients with a germline genetic susceptibility.

## 2. Results

### 2.1. Patients’ Characteristics of the Cohort

A total of 6597 patients were included in the study and were assessed with multi-gene panel testing due to HBOC-related cancers. These female HBOC patients included 5594 patients with breast cancer, 639 patients with ovarian cancer and 241 patients with both breast and ovarian cancers. All male patients were diagnosed with male breast cancer. The median age at diagnosis of breast cancer was 46.6 years (range 18–95). Among these patients, bilateral breast cancers were observed in 1246 patients (20.9%). Most breast cancers were ductal carcinoma (NOS type) (*N* = 5087; 71.9%). A high percentage of breast cancers were found to be hormone-receptor-positive (*N* = 3997; 74.1%) and with 17.9% TNBC (*N* = 966). Most of the breast tumor grading favored grades 2 or 3 (*N* = 2191; 45.2% and *N* = 1867; 38.5%, respectively). A positive family history of *BRCA-related* cancer was also noted in 3971 (60.2%) of their relatives. A family history of breast cancer (in first or second-degree relatives) was observed in 2663 patients (40.4%). Detailed clinicopathological characteristics are shown in Table 1.

**Table 1:** Clinicopathological characteristics of our cohort.

	Beyond *BRCA* *N* = 234		*BRCA1/2* *N* = 718		MINAS *N* = 20		Total *N* = 6597		*p*-Value (Beyond *BRCA* vs. *BRCA1/2*)
	*N*	%	*N*	%	*N*	%	*N*	%	
Gender									
F	232	99.1%	699	97.4%	20	100.0%	6475	98.2%	0.127
M	2	0.9%	19	2.6%	0	0.0%	122	1.8%	
Personal History of Breast and/or OV Cancers (Female Only) ^									
Breast	195	84.4%	494	70.7%	16	80.0%	5594	86.4%	<0.001
Breast & Ovarian	14	6.1%	86	12.3%	4	20.0%	241	3.7%	
Ovarian	22	9.5%	119	17.0%	0	0.0%	639	9.9%	
Breast CA First DX									
Mean	44.3		43.2		42.8		46.6		0.212
Median	43		42		38.5		44		0.561
SD	12.4		10.2		11.2		11.9		
Range	18–81		21–84		30–69		18–95		
Bilateral Breast Cancers									
Y	62	29.4%	178	29.7%	9	45.0%	1246	20.9%	1
N	149	70.6%	421	70.3%	11	55.0%	4711	79.1%	
Personal Multiple Cancers									
Y	59	25.2%	237	33.0%	5	25.0%	1236	18.7%	0.028
N	175	74.8%	481	67.0%	15	75.0%	5361	81.3%	
Personal Other Cancer									
Breast	211	90.2%	598	83.3%	20	100.0%	5954	90.3%	0.011
Ovarian	28	12.1%	160	22.9%	3	15.0%	751	11.6%	<0.001
Peritoneal	0	0.0%	22	3.1%	0	0.0%	53	0.8%	0.004
Fallopian Tube	0	0.0%	20	2.9%	0	0.0%	40	0.6%	0.007
Uterus	16	6.9%	12	1.7%	1	5.0%	157	2.4%	<0.001
Colorectal	7	3.0%	8	1.1%	0	0.0%	66	1.0%	0.065
Bladder	0	0.0%	4	0.6%	0	0.0%	10	0.2%	0.577
Liver	0	0.0%	4	0.6%	1	5.0%	15	0.2%	0.577
Pancreatic	0	0.0%	1	0.1%	0	0.0%	14	0.2%	1.000
Family History of Cancers (1st & 2nd Degree)									
Breast	102	43.6%	422	58.8%	9	45.0%	2663	40.4%	<0.001
Ovarian	12	5.1%	130	18.1%	5	25.0%	371	5.6%	<0.001
Lung	57	24.4%	188	26.2%	8	40.0%	1453	22.0%	0.606
Colorectal	61	26.1%	145	20.2%	3	15.0%	1210	18.3%	0.067
Liver	26	11.1%	100	13.9%	1	5.0%	793	12.0%	0.317
Stomach	26	11.1%	88	12.3%	2	10.0%	583	8.8%	0.728
NPC	13	5.6%	74	10.3%	0	0.0%	599	9.1%	0.027
Pancreatic	14	6.0%	63	8.8%	0	0.0%	348	5.3%	0.214
BRCA-related	155	66.2%	551	76.7%	14	70.0%	3971	60.2%	0.002
**Breast Cancers**	**N = 273**		**N = 777**		**N = 29**		**N = 7203**		
Histology									
Ductal	203	75.5%	592	78.5%	27	93.1%	5087	71.9%	0.258
In situ	41	15.2%	86	11.4%	1	3.4%	1192	16.9%	
Others	25	9.3%	76	10.1%	1	3.4%	793	11.2%	
NS	4		23		0		131		
Grade									
1	16	8.3%	25	4.6%	2	8.3%	787	16.2%	0.003
2	86	44.8%	189	35.0%	8	33.3%	2191	45.2%	
3	90	46.9%	326	60.4%	14	58.3%	1867	38.5%	
NS	40		151		4		1166		
TNBC									
Y	37	16.8%	219	34.2%	7	26.9%	966	17.1%	<0.001
N	183	83.2%	421	65.8%	19	73.1%	4699	82.9%	
NS	12		51		2		346		
Hormonal Pos									
Y	167	75.6%	400	61.2%	19	73.1%	4264	74.5%	<0.001
N	54	24.4%	254	38.8%	7	26.9%	1457	25.5%	
NS	11		37		2		290		
Her2									
Pos	50	21.2%	69	10.3%	4	14.8%	1306	21.0%	<0.001
Equivocal	17	7.2%	37	5.5%	0	0.0%	361	5.8%	
Neg	169	71.6%	565	84.2%	23	85.2%	4539	73.1%	
NS	37		106		2		997		
**OV cancers**	***N*** ** = 36**		***N*** ** = 205**		***N*** ** = 4**		***N*** ** = 880**		
Ovarian CA First DX									
Mean	45.2		53.3		49.3		49.2		<0.001
Median	44		52		45.5		49		
SD	9.9		9.8		9.5		11.4		
Range	29–65		17–85		43–63		9–85		
Sites									
Ovarian	28	77.8%	149	72.7%	3	75.0%	723	82.7%	<0.001
Fallopian Tube	0	0.0%	21	10.2%	0	0.0%	36	4.1%	
Peritoneal	0	0.0%	20	9.8%	0	0.0%	50	5.7%	
Uterus	7	19.4%	6	2.9%	1	25.0%	45	5.1%	
Mixed	1	2.8%	9	4.4%	0	0.0%	20	2.3%	
NS	0		0		0		6		
Histological Type									
Epithelial	24	88.9%	176	95.7%	2	100.0%	753	95.6%	0.026
Germ Cell	0	0.0%	1	0.5%	0	0.0%	9	1.1%	
Stromal	1	3.7%	0	0.0%	0	0.0%	8	1.0%	
Others	2	7.4%	1	0.5%	0	0.0%	4	0.5%	
Mixed	0	0%	6	3.3%	0	0.0%	14	1.8%	
NS	9		21		2		92		
Grade									
1	3	12.5%	1	0.6%	0	0.0%	76	10.4%	0.004
2	2	8.3%	4	2.3%	0	0.0%	152	20.7%	
3	19	79.2%	165	93.8%	3	100.0%	491	67.0%	
Mixed	0	0%	6	3.4%	0	0.0%	14	1.9%	
NS	12		29		1		147		

^ An *NF1* carrier with uterine corpus cancer was excluded.

### 2.2. Summary of Mutations Identified

Nine hundred and seventy-two HBOC patients (14.7%) carried at least one P/LP mutation. A total of 952 (14.4%) patients had one P/LP variant from NCCN-listed genes and 20 (0.3%) patients carried more than one mutation. Among those patients only carrying one P/LP mutation, mutations in *BRCA1/2* and non-*BRCA* cancer susceptibility genes accounted for 10.9% (*N* = 718) and 3.5% (*N* = 234) respectively. Apart from *BRCA1* (*N* = 346; 36.3%) and *BRCA2* (*N* = 372; 39.1%), *PALB2* was the most commonly identified gene with P/LP variants in 88 individuals (9.2%), followed by *TP53* (*N* = 28, 2.9%), *ATM* (*N* = 17, 1.8%) and *BARD1* (*N* = 17, 1.8%). Deficient mismatch repair (MMR) genes (*MLH1*, *MSH2*, *MSH6* and *PMS2*) accounted for 2.1% (*N*  =  20). Details of P/LP variants are shown in Table 2 and Supplementary Table S1.

One thousand six hundred and thirty-three family members of mutation carriers also underwent genetic screening on their specific mutation; among these family members, 47.5% (*N* = 777) of them also carried the same mutation variants. A total of 647 of them are *BRCA* carriers, while 131 are carriers of beyond BRCA and four of them carried double mutations.

### 2.3. BRCA1/2 Carriers

From a total of 718 HBOC cancer patients with *BRCA1/2* P/LP variants, *N* = 646 (90.0%) fulfilled at least one of the 2026 NCCN testing criteria for high-penetrance breast cancer susceptibility genes. The most common indication was early-onset breast cancer (*N* =  481, 67.0%), followed by family histories (*N*  =  427, 59.5%) including young breast CA (*N* = 304, 50.8%), male breast CA (*N* = 11, 1.8%), ovarian CA (*N* = 103, 21.7%), pancreatic CA (*N* = 63, 10.5%), and metastatic or high-risk prostate CA (*N* = 55, 9.2%). Besides family history, these carriers included patients with triple-negative breast cancer (*N*  =  187, 26.0%). Others were ipsilateral or contralateral breast cancers (*N*  =  178, 24.8%) and male breast cancer (*N*  =  19, 2.7%). The proportion of patients with P/LP variants in *BRCA1/2* who met each criterion is shown in Table 3. Among these *BRCA1/2* mutation carriers, 79.0% of these carries fulfilled one to three of these NCCN criteria. The detailed number of criteria met by carriers is shown in Table 4.

### 2.4. Beyond BRCA1/2 Carriers with Breast Cancer

From a total of 234 HBOC cancer patients with P/LP variants, 127 carriers (54.3%) had a mutation from high-penetrance genes (*CDH1*, *PALB2*, *PTEN* and *TP53*) while 107 (45.7%) were moderate- to low-penetrance gene carriers. Among these carriers, *N* = 211 (90.2%) fulfilled the 2026 NCCN testing criteria for high-penetrance breast cancer susceptibility genes. The most common indication was early-onset breast cancer (*N*  =  158, 67.5%), followed by family histories (*N*  =  83, 35.5%), including family histories of young breast CA (*N*  =  54, 25.6%), male breast CA (*N*  =  2, 0.9%), ovarian CA (*N*  =  12, 5.7%), pancreatic CA (*N*  =  14, 6.6%), and metastatic or high-risk prostate CA (*N*  =  66, 4.0%). Certain majorities were ipsilateral or contralateral breast cancers (*N*  =  62, 26.5%) and triple-negative breast cancer (*N*  =  35, 15.0%). The proportion of patients with P/LP variants in beyond *BRCA* genes who met each criterion is shown in Table 3. Among these beyond *BRCA1/2* mutation carriers, 72.2% of these carriers fulfilled one to two of these NCCN criteria. The detailed number of criteria met by carriers is shown in Table 4.

### 2.5. Beyond BRCA1/2 Carriers with Ovarian Cancer

There were 880 patients with a personal history of ovarian cancers in our HBOC cohort. Their median age of diagnosis of ovarian cancer was 49, ranging from 9 to 85. Of 241 ovarian cancer patients with germline P/LP variants, 205 (23.3%) were *BRCA1/2* carriers while only 36 (4.1%) were beyond *BRCA* carriers (Table 1). *PALB2* was the most commonly identified gene with P/LP variants in nine individuals (1.0%). Deficient mismatch repair (MMR) genes (*MLH1*, *MSH2*, *MSH6* and *PMS2*) accounted for 1.3% (*N*  =  11) of patients with ovarian cancer. Details of P/LP variants are shown in Table 2.

### 2.6. Multi-Locus Inherited Neoplasia Allele Syndrome (MINAS)

In our cohort, there were twenty individuals identified with double P/LP variants. Thirteen patients had concomitant non-*BRCA* and *BRCA1* or *BRCA2* P/LP variants. One patient found double pathogenic *BRCA1* variants and one patient found triple mutations from *ATM*, *BRCA1* and *CHEK2*. Details of these MINAS patients are listed in Table 5. The predispositions of MINAS cases have been discussed previously [7].

### 2.7. Surveillance Outcomes for Beyond BRCA Mutation Carriers

Management strategies were discussed with 192 female mutation carriers, comprising both breast cancer probands (*N* = 132) and their high-risk unaffected relatives (*N* = 60). Among this cohort, 12% of the carriers (22 probands and one family member) have already undergone bilateral mastectomies, while 169 carriers opted for annual surveillance breast screening. Within the surveillance group, *PALB2* mutation carriers (*N* = 108) represented the majority (probands *N* = 62; family members *N* = 46); 63.9%), with mean and median follow-up periods of 3.1 years. During the surveillance interval, seven new primary breast cancers and two recurrences were identified among *PALB2* carriers, yielding an overall cancer detection rate of 8.3%. For the *TP53* cohort *N* = 17 (probands *N* = 13; family members *N* = 4), the mean and median follow-up durations were 6.3 and 6.8 years, respectively. In this group, three new primary breast cancers and two recurrences were detected from proband carriers, resulting in an overall detection rate of 29.4%. Among the six *PTEN* mutation proband carriers managed at our clinic (mean follow-up: 4.2 years; median: 3.2 years), two patients (33.3%) were diagnosed with new primary breast cancers. An additional 38 carriers of other non-*BRCA* mutations were also managed, though their surveillance duration was limited (mean/median follow-up: 1.0–1.5 years). Within this subset, which primarily consisted of *BARD1* mutation carriers (*N* = 18, probands *N* = 12; family members *N* = 6), only one new breast cancer was detected from proband. High-risk surveillance outcomes for *BRCA1/2* mutation carriers in our population have been described previously [8].

## 3. Discussion

Germline *BRCA1* and *BRCA2* testing for patients with breast cancer and ovarian cancer has been integrated into many national healthcare systems worldwide, including Hong Kong. In Hong Kong, the Hospital Authority, the main body for public health, now offers germline *BRCA1/2* testing to all newly diagnosed ovarian cancer patients and invasive breast cancer patients; the approach supports identification of individuals who may benefit from targeted therapeutic options, such as PARP inhibitors. While this represents a crucial step toward personalized oncology care, the current system may still be less oriented toward enabling patients to act on cancer predisposition through oncological prevention and risk-adapted diagnostics.

Importantly, effective cancer prevention should begin with identifying individuals at high risk of developing cancer. Hereditary breast and ovarian cancer (HBOC) represents a key high-risk subgroup, and this study specifically focuses on an HBOC cohort because affected individuals have a higher chance of developing cancer over their lifetime. By characterizing HBOC-associated pathogenic and likely pathogenic (P/LP) variants, risk stratification can be used to inform prevention strategies, surveillance, and early detection for both patients and potentially affected family members.

In the 2020s, advances in high-throughput NGS have made testing multiple HBOC genes an international standard for hereditary breast and ovarian cancer evaluation. Clinical practice guidelines from the U.S. National Comprehensive Cancer Network (NCCN) also recommend expanding genetic testing, not only limiting it to *BRCA1/2* but including other HBOC-related genes [9]. Consistent with this rationale, extending hereditary testing to beyond *BRCA1/2* may be important for HBOC patients because P/LP variants are not confined to a single gene. In our cohort, mutations in *BRCA1/2* accounted for 10.9% (*N* = 718) of patients, whereas non-*BRCA* cancer susceptibility genes accounted for 3.5% (*N* = 234). Among these non-*BRCA* cancer susceptibility genes, mutations from high-penetrance genes (*CDH1*, *PALB2*, *PTEN* and *TP53*) have been identified in 54.3% of cases (*N* = 127) while 107 (45.7%) were moderate- to low-penetrance gene (*ATM*, *CHEK2*, *BARD1*, *RAD51C/D* and others) mutation carriers. It is interesting that there was a relatively large number of *RAD51C/D* carriers, and the penetrance and phenotypic characteristics of these Chinese carriers have been specifically discussed previously [10]. Overall, these findings suggest that clinically actionable hereditary risk may be missed when testing is limited to *BRCA1/2* alone. Multi-gene panel testing may therefore provide a more comprehensive genetic landscape of HBOC and can support more accurate risk assessment, tailored surveillance and risk-reducing strategies, and informed family counseling, ultimately improving precision in prevention and management.

However, in Hong Kong, policies for subsidizing non-*BRCA* gene testing still vary, and broader hereditary testing has not yet been fully incorporated into public medical coverage. Instead, multigene testing beyond *BRCA1/2* has been largely supported through the Hong Kong Hereditary Breast Cancer Family Registry, which is charity-funded, whereas preventive surgeries for selective individuals remain primarily covered by the Hospital Authority. Selective individuals may benefit from partially funded breast surveillance under the Health Bureau of the HKSAR, subject to resource availability, whereas a large portion of breast surveillance is still funded by the Hong Kong Hereditary Breast Cancer Registry.

In view of the landscape of HBOC management in Asia, Singapore, South Korea and Taiwan have increasingly integrated multigene panel testing into public health frameworks. As of March 2026, the Singapore Ministry of Health (MOH) has formalized a comprehensive framework to transition from testing *BRCA1/2* only to standard coverage for approximately 25–44 genes [11]. Full implementation was scheduled for December 2026, and the initiative provides means-tested subsidies of up to 70% for eligible citizens through residents’ mandatory national savings scheme, together with universal health insurance. This model is intended to facilitate not only germline testing, but also risk-reducing mastectomy (RRM) and bilateral salpingo-oophorectomy (RRBSO). Following the introduction of subsidies, the uptake of genetic testing in Singapore increased by approximately 20% [12]. This suggests that barriers to service utilization extend beyond technical availability and include system-level factors such as referral pathways, risk awareness, and cost [13].

Similarly, South Korea has adopted a robust genomic strategy through its National Health Insurance (NHI) system, which has provided conditional coverage for genetic testing since 2017 [14]. Their testing standard has focused on clinically actionable genes and included 14 high-to-moderate penetrance genes, such as *PALB2*, *TP53*, *PTEN*, *ATM*, *CHEK2*, *RAD51C*, and *RAD51D*, with government subsidies projected to reach approximately 70% by late 2026 for high-risk cohorts [15]. A distinguishing feature of the Korean model is its longitudinal care continuum, which supports both prophylactic interventions and intensive surveillance, including annual breast MRI.

In contrast, Japan has a well-established clinical infrastructure governed by the Ministry of Health, Labor and Welfare (MHLW) and the Japanese Organization of Hereditary Breast and Ovarian Cancer (JOHBOC), but reimbursement has historically been more concentrated on *BRCA1/2* for companion diagnostics and preventive management. Broader multigene panels are often associated with out-of-pocket expenditures or may be limited to investigative clinical trials. Nevertheless, risk-reducing procedures such as RRM and RRBSO for confirmed mutation carriers with prior cancer are covered by national insurance, and surveillance screenings including annual breast MRI can also be reimbursed for confirmed mutation carriers [16]. These differences highlight that reimbursement policies for testing and for downstream prevention/surveillance are intertwined and should be considered together.

Taiwan has also shifted toward precision oncology. As of May 2024, Taiwan’s National Health Insurance (NHI) began covering NGS panels for 19 cancer types, including triple-negative breast cancer (TNBC) and ovarian cancer. This policy represents a move toward a “beyond *BRCA*” approach by providing fixed-amount reimbursements for genetic testing beyond *BRCA1/2*. Under this framework, the NHI offers a subsidy to cover part of the costs of more comprehensive multigene panels, and patients may choose panel size (ranging from 2 to more than 100 genes) depending on financial status and needs [17]. However, risk-reducing surgeries and high-intensity surveillance are not yet universally reimbursed as standard preventive items within the NHI package. As a result, carriers may need to pay out of pocket for MRI surveillance or rely on hospital-specific research programs [18].

The relative progress in these higher-income jurisdictions contrasts with the more constrained frameworks in Thailand and Malaysia. Although Thailand implemented universal reimbursement for germline *BRCA1/2* testing in 2022, expanded multigene testing and prophylactic interventions for asymptomatic carriers remain largely uncovered by public schemes [19]. Malaysia faces even greater challenges, where predisposition testing remains predominantly out-of-pocket due to limited national budget allocation and the exclusion of genetic screening from private medical insurance [19].

To ensure Hong Kong does not fall behind other regional programs, policy discussions may consider moving genetic testing toward clinically actionable beyond *BRCA* multigene panels for HBOC. Singapore and South Korea have already recognized that genes such as *PALB2*, *RAD51C*, *RAD51D*, and *ATM* can meaningfully refine risk assessment and guide risk-adapted management, supported by reimbursement frameworks that improve access. A practical compromise for Hong Kong could be government-supported NGS panels combined with structured co-payment or means-tested subsidies to expand access while limiting the financial burden on public resources. Importantly, genetic programs only reduce cancer mortality when downstream care is also supported. With increased evidence of the mortality impact of surveillance and preventative measures, such interventions should not be limited only to those related to breast cancers; a spectrum of hereditary cancers related to specific gene mutation should also be integrated into care pathways rather than treated as separate and unaffordable expenditures to increase the prognostic impact of genetic testing. The increasing support in the literature for the cost-effectiveness of risk-reducing and preventative strategies could provide a good reference for policy makers for more advanced planning of genetic services. Finally, strengthening protections against genetic discrimination, particularly related to medical insurance coverage, may help reduce deterrence to testing and may encourage participation among high-risk individuals.

## 4. Materials and Methods

### 4.1. Participants and Selection Criteria

We recruited 6597 HBOC patients with breast and ovarian cancers from the Hong Kong Hereditary Breast Cancer Family Registry using the following inclusion criteria: (1) at least one first- or second-degree relative with *BRCA*-associated cancer, regardless of age; (2) diagnosis of breast cancer at age 45 or younger; (3) bilateral breast cancer; (4) triple negative or ER+/Her2- breast cancers; (5) from a *BRCA* mutation-related family; (6) male breast cancer; (7) ovarian cancer. We also invited 1635 family members from these mutation carriers for genetic tests. Clinical and pathological data for these patients were extracted from their medical records (see Table 1). All participants gave written informed consent, and the study was conducted in accordance with the Declaration of Helsinki. Pre- and post-test genetic counseling services were administered to patients. Comprehensive care including variant interpretation, psychosocial support, oncological risk quantification, and the coordination of relevant clinical management was offered.

### 4.2. Multi-Gene Panel Testing by NGS

Genomic DNA were extracted from patient’s peripheral blood and subjected to multi-gene sequencing analysis using next-generation sequencing (NGS). The gene panel was changed from time to time due to technological advancement; however, all patients in this cohort at least sequenced the following NCCN’s manageable HBOC genes (NCCN Genetic/Familial High-Risk Assessment: Breast, Ovarian, Pancreatic, and Prostate v3.2026): *ATM*, *BARD1*, *BRCA1*, *BRCA2*, *BRIP1*, *CDH1*, *CDKN2A*, *CHEK2*, *MSH2*, *MLH1*, *MSH6*, *PMS2*, *EPCAM*, *NF1*, *PALB2*, *PTEN*, *RAD51C*, *RAD51D*, *STK11*, and *TP53*. Library preparation, sequencing, bioinformatics, variant interpretation, annotation, and statistical analysis were conducted as previously described [20]. Paired-end reads were aligned to the human reference genome GRCh37/hg19. Variants with a minor allele frequency ≥1% in the 1000 Genomes Project [21] were excluded from manual curation. Variant nomenclature followed Human Genome Variation Society (HGVS) guidelines and was checked with LUMC Mutalyzer 3 (http://mutalyzer.nl, accessed 13 April 2026). To assess NGS performance and accuracy, a known *BRCA1/2* positive control was included in each run, and orthogonal confirmations were done on every P/LP variant, including short variants and large structural rearrangements [20].

### 4.3. Breast Surveillance for Beyond BRCA Mutation Carriers

Confirmed mutation carriers were managed within a multidisciplinary high-risk clinic by specialized clinicians and genetic counselors. Management strategies encompassed risk-reducing mastectomy (RRM), with or without immediate breast reconstruction (IBR), risk-reducing bilateral salpingo-oophorectomy (RRBSO), chemoprevention, and intensive surveillance. The surveillance protocol consisted of biannual clinical breast examinations and annual imaging (mammography/digital breast tomosynthesis with ultrasound), supplemented by annual contrast-enhanced breast MRI, with imaging modalities alternated every six months.

### 4.4. Statistical Analysis

Fisher’s exact test was used to assess the association between selection variables and mutation status. A *p*-value below 0.05 was considered statistically significant for all analyses. Statistical analyses were conducted using R (version 3.4.2) [22].

## 5. Conclusions

In this multi-gene hereditary cancer cohort, germline pathogenic or likely pathogenic mutations were identified in 10.9% of cases for *BRCA1/2* and 3.5% for non-*BRCA* susceptibility genes. These beyond *BRCA* variants represent a substantial share of actionable hereditary cancer risk. *PALB2* was the most frequently identified beyond *BRCA* gene, followed by *TP53*, *ATM*, and *BARD1*. Our findings suggest that clinically actionable hereditary predisposition may extend beyond the scope of *BRCA1/2*. Although this study does not directly measure patient outcomes, the data may inform a strategic refinement of prevention-oriented policies in Hong Kong, specifically providing a rationale for strengthening public healthcare pathways for multi-gene panel testing and for further translating genetic results into risk-adapted clinical management. By aligning genomic diagnostics with tailored surveillance and risk-reducing interventions, hereditary cancer programs may move beyond risk identification to measurable improvements in real-world patient outcomes, although this would require prospective evaluation.

## Figures and Tables

**Table 2:** Identification of Pathogenic/Likely Pathogenic Variants in Actionable Hereditary Cancer Genes.

Gene		Total (*N* = 6597)		Breast Cancer (*N* = 5594)		Ovarian Cancer (*N* = 639)		Breast and Ovarian Cancers (*N* = 241)	
		*N*	%	*N*	%	*N*	%	*N*	%
*BRCA1/2*	*BRCA1*	346	5.2%	209	3.7%	84	13.1%	53	22%
	*BRCA2*	372	5.6%	304	5.4%	35	5.5%	33	13.7%
Beyond *BRCA1/2*	*ATM*	17	0.3%	16	0.3%	0	0%	1	0.4%
	*BARD1*	17	0.3%	16	0.3%	1	0.2%	0	0%
	*BRIP1*	9	0.1%	8	0.1%	0	0%	1	0.4%
	*CDH1*	1	0%	1	0%	0	0%	0	0%
	*CHEK2*	14	0.2%	14	0.3%	0	0%	0	0%
	*MLH1*	1	0%	1	0%	0	0%	0	0%
	*MSH2*	10	0.2%	2	0%	6	0.9%	2	0.8%
	*MSH6*	8	0.1%	5	0.1%	3	0.5%	0	0%
	*NF1*	3	0%	2	0%	1	0.2%	0	0%
	*PALB2*	88	1.3%	79	1.4%	5	0.8%	4	1.7%
	*PMS2*	1	0%	1	0%	0	0%	0	0%
	*PTEN*	10	0.2%	6	0.1%	1	0.2%	3	1.2%
	*RAD51C*	12	0.2%	8	0.1%	3	0.5%	1	0.4%
	*RAD51D*	15	0.2%	12	0.2%	3	0.5%	0	0%
	*TP53*	28	0.4%	26	0.5%	0	0%	2	0.8%
Negative		5625	85.3%	4884	87.3%	497	77.8%	141	58.5%

**Table 3:** HBOC patients fulfilling the NCCN v3.2026 testing guideline.

	Beyond *BRCA* Carriers *N* = 234		*BRCA1/2* Carriers *N* = 718		MINAS Carriers *N* = 20		Total *N* = 6597	
	*N*	%	*N*	%	*N*	%	*N*	%
Breast cancer Dx ≤ 50 years	158	67.5%	481	67%	16	80%	4106	62.2%
Ipsilateral or contralateral breast	62	26.5%	178	24.8%	9	45%	1246	18.9%
TNBC	35	15%	187	26%	7	35%	924	14%
Male breast	2	0.9%	19	2.6%	0	0%	122	1.8%
Lobular breast cancer with personal or FH	10	4.3%	19	2.6%	0	0%	248	3.8%
Eligibility for PARP inhibitor therapy	28	12%	87	12.1%	3	15%	589	8.9%
≥1 FH of young breast, male breast, ovarian, pancreatic CA, met/high risk prostate cancer	83	35.5%	427	59.5%	11	55%	2119	32.1%
≥3 FH of breast or prostate cancer	13	5.6%	77	10.7%	1	5%	225	3.4%

FH = Family History.

**Table 4:** Number of criteria met by our HBOC cohort.

	*BRCA1/2*	Beyond BRCA (NCCN)	MINAS	Other Genes (Non-NCCN)	Negative
*N*	718	234	20	118	5507
Criteria = 0	72	23	0	13	831
%	10.0%	9.8%	0.0%	11.0%	15.1%
Criteria = 1	169	89	4	55	2510
%	23.5%	38.0%	20.0%	46.6%	45.6%
Criteria = 2	217	80	8	39	1685
%	30.2%	34.2%	40.0%	33.1%	30.6%
Criteria = 3	181	29	6	10	411
%	25.2%	12.4%	30.0%	8.5%	7.5%
Criteria = 4	67	12	1	0	66
%	9.3%	5.1%	5.0%	0.0%	1.2%
Criteria = 5	11	1	1	1	4
%	1.5%	0.4%	5.0%	0.9%	0.1%
Criteria = 6	1	0	0	0	0
%	0.1%	0.0%	0.0%	0.0%	0.0%

**Table 5:** Characteristics of MINAS carriers.

Patient ID	Mutations	Personal Cancers		Family History	
MINAS_01	*BRCA1*: c.4065_4068delTCAA; p.Asn1355Lysfs*10 *BRCA1*: c.5406+7A>G; r.5333_5406del74; p.Asp1778Glyfs*27	44 43	Breast Cancer Ovarian Cancer	55 52 45 35	Sister: Breast & Ovarian Cancers Mother: Cervical Cancer Sister: Cervical Cancer
MINAS_02	*BRCA1*: c.470_471delCT; p.Ser157* BARD1: c.539_540delAT; p.Tyr180*	39	Breast Cancer		No FH of Cancer
MINAS_03	*BRCA1*: c.3531dupT; p.Ser1178* CHEK2: c.1461+1G>A; r.1376_1461del86; p.Ala459Glyfs*2	63 69 72	Ovarian Cancer Breast Cancer Liver Cancer	49 49 36 55 42 66	Daughter: Breast & Endometrial Cancers Brother’s Daughter: Breast Cancer Mother: Lymphoma Sister: Ovarian Cancer Brother: Gallbladder Cancer
MINAS_04	*BRCA1*: c.876_879delCACT; p.Thr293Lysfs*4 *CHEK2*: c.1315C>T; p.Gln439* ATM: Deletion of exons 41-45; c.6006+1_6007-1_6572+1_6573-1del; r.6007_6572del; p.Asp2003Ilefs*5	35	Breast Cancer	UK UK	Father: Liver Cancer Father’s Brother: Lung Cancer
MINAS_05	*BRCA1*: c.505C>T; p.Gln169* GEN1: c.2708dupT; p.Arg904LysfsTer36	37	Breast Cancer	75 58	Father: Lung Cancer Mother: Ovarian & Peritoneal Cancers
MINAS_06	*BRCA1*: c.3342_3345delAGAA; p.Glu1115* MSH6: c.3013C>T; p.Arg1005*	34	Breast Cancer	47 36	Mother’s Brother: Laryngeal Cancer Mother’s Sister’s Son: Lung Cancer
MINAS_07	*BRCA1*: c.3286C>T; p.Gln1096* PALB2: c.857delC; p.Pro286Leufs*2	30 32 48 58 67	Breast Cancer Breast Cancer Ovarian Cancer Laryngeal Cancer Lymphoma	61 44 UK UK UK	Sister: Breast Cancer Sister: Breast Cancer Father’s Mother: Uterus & Ovarian Cancers Father’s Sister: Ovarian Cancer Father’s Sister’s Daughter: Ovarian & Breast Cancers
MINAS_08	*BRCA2*: c.262_263delCT; p.Leu88Alafs*12 *ATM*: c.7515+1G>T; r.7308_7515del208; p.Tyr2437Glufs*4	33	Breast Cancer	17	Mother’s Sister’s Daughter: Cancer of Unknown Primary
MINAS_09	*BRCA1*: c.5511G>C; p.Trp1837Cys *BRCA2*: c.7471C>T; p.Gln2491*	38 38	Breast Cancer Breast Cancer	39 41 50 55 55 45 64 UK UK	Sister: Breast Cancer Sister: Breast & Lung Cancers Father’s Mother: Bilateral Breast Cancers Father’s Sister: Breast Cancer Mother’s Sister: Breast Cancer Father’s Father: Lung Cancer Mother’s Father: Prostate Cancer
MINAS_10	*BRCA2*: c.3109C>T; p.Gln1037* HERC1 ^: c.363C>A; p.Tyr121*	50	Breast Cancer	47 48 25 74 45 75	Sister: Bilateral Breast Cancers Sister’s Daughter: Breast Cancer Father: Stomach Cancer Brother: Colorectal Cancer Father’s Brother: Stomach Cancer
MINAS_11	*BRCA2*: c.7480C>T; p.Arg2494* NF1: c.2970_2972del; p.Met992del	31	Breast Cancer	30 50	Mother: Breast Cancer Mother’s Sister: Breast Cancer
MINAS_12	*BRCA2*: c.7379_7382delACAA; p.Asn2460Thrfs*8 *RAD51C*: c.394dupA; p.Thr132Asnfs*23	57 50	Breast Cancer Breast Cancer	78 63 62 55	Father: Lung Cancer Brother: Esophageal & Prostate Cancers Mother’s Brother’s Son: Lung Cancer
MINAS_13	*BRCA2*: c.2442delC; p.Met815Trpfs*10 *PTEN*: c.697C>T; p.Arg233*	58 58	Breast Cancer Breast Cancer	40	Father: Breast Cancer
MINAS_14	*BRCA1*: c.5089T>C; p.Cys1697Arg *TP53*: c.541C>T; p.Arg181Cys	36 42	Breast Cancer Breast Cancer	57 85 UK	Father: Lung Cancer Mother’s Father: Testicular Cancer Father’s Sister: Ovarian Cancer
MINAS_15	*BRIP1*: c.1A>G *MSH6*: c.3913_3931dup19; p.Glu1311Alafs*3	57	Breast Cancer	75 79 80	Father: Colorectal Cancer Mother: Colorectal & Uterus Cancers
MINAS_16	*BRIP1*: c.2392C>T; p.Arg798* TP53: c.529_546del; p.Pro177_Cys182del	30 34 37	Breast Cancer Breast Cancer Lung Cancer	UK 73 53 50 59	Father’s Mother: Stomach & Lung Cancers Father’s Brother: Laryngeal Cancer Father’s Brother: Lung Cancer Mother’s Brother: Prostate Cancer
MINAS_17	*CHEK2*: c.1072C>T; p.Gln358* MAP3K1: c.2347C>T; p.Gln783*	44 44	Breast Cancer Breast Cancer	65 60 63 68	Mother: Breast Cancer Father: Kidney & Colorectal Cancers Mother’s Father: Lung Cancer
MINAS_18	*PALB2*: c.1048C>T; p.Gln350* RB1: c.1468G>A; p.Ala490Thr	36	Breast Cancer	59 60 55 UK UK	Mother: Breast Cancer Father’s Sister: Breast Cancer Mother’s Sister: Breast Cancer Mother’s Father: Cancer of Unknown Primary Mother’s Brother: Cancer of Unknown Primary
MINAS_19	*PTEN*: c.697C>T; p.Arg233* NF1: c.1885G>C; p.Gly629Arg	58 58 43	Breast Cancer Breast Cancer Uterus Cancer	59	Mother’s Sister Daughter: Breast Cancer
MINAS_20	*BRIP1*: c.1343G>A; p.Trp448* RAD51D: c.480+2T>G	46 49	Breast Cancer Breast Cancer	56	Father: Lung Cancer

^ *HERC1* has been included due to its relationship with other critical breast cancer pathways, such as HER-2 signaling or homologous recombination repair (HRR) deficiencies.

## Data Availability

The dataset supporting the conclusions of this article is included within the article.

## Supplementary Materials

The following supporting information can be downloaded at: https://www.mdpi.com/article/10.3390/ijms27177572/s1.
