# Supplementary material for: Genomic Landscape of 6597 Hong Kong HBOC Patients: Implications for Beyond-BRCA Multi-Gene Panel Testing and Cancer Surveillance
Source: Int J Mol Sci. 2026 Aug 24;27(17):7572. doi: 10.3390/ijms27177572 (PMC13566287; doi:10.3390/ijms27177572)
Supplement: Supplementary file 1 [file ijms-27-07572-s001.zip › ijms-4501572-supplementary.pdf]

Supplementary Table S1. Clinicopathological characteristics of beyond BRCA carriers.

	ATM **N=17**		***BARD1*** **N=17**		***PALB2*** **N=88**		***TP53*** **N=28**	
	**N**	**%**	**N**	**%**	**N**	**%**	**N**	**%**
Gender								
F	17	100.0%	17	100.0%	87	98.9%	28	100.0%
M	0	0.0%	0	0.0%	1	1.1%	0	0.0%
Personal History of Breast and/or OV cancers (Female only)								
Breast	16	94.1%	16	94.1%	78	89.7%	26	92.9%
Breast & Ovarian	1	5.9%	0	0.0%	4	4.6%	2	7.1%
Ovarian	0	0.0%	1	5.9%	5	5.7%	0	0.0%
Breast CA First DX								
Mean	45.1		45.1		44.7		37.2	
Median	45.0		45.0		43.0		33.0	
SD	11.1		11.1		10.8		15.2	
Range	30-62		30-62		24-69		18-81	
Bilateral Breast Cancers								
Y	3	17.6%	2	12.5%	25	30.1%	13	46.4%
N	14	82.4%	14	87.5%	58	69.9%	15	53.6%
Personal Multiple Cancers								
Y	4	23.5%	2	11.8%	14	15.9%	10	35.7%
N	13	76.5%	15	88.2%	74	84.1%	18	64.3%
Personal Other Cancer								
Breast	17	100.0%	16	94.1%	83	94.3%	28	100.0%
Ovarian	1	5.9%	1	5.9%	6	6.9%	2	7.1%
Peritoneal	0	0.0%	0	0.0%	0	0.0%	0	0.0%
Fallopian tube	0	0.0%	0	0.0%	0	0.0%	0	0.0%
Uterus	0	0.0%	1	5.9%	3	3.4%	0	0.0%
Colorectal	2	11.8%	0	0.0%	1	1.1%	0	0.0%
Bladder	0	0.0%	0	0.0%	0	0.0%	0	0.0%
Liver	0	0.0%	0	0.0%	0	0.0%	0	0.0%
Pancreatic	0	0.0%	0	0.0%	0	0.0%	0	0.0%
Family History of Cancers (1st & 2nd degree)								
Breast	7	41.2%	7	41.2%	48	54.5%	9	32.1%
Ovarian	1	5.9%	1	5.9%	2	2.3%	0	0.0%
Lung	2	11.8%	4	23.5%	23	26.1%	8	28.6%
Colorectal	3	17.6%	5	29.4%	18	20.5%	10	35.7%

Liver	1	5.9%	3	17.6%	13	14.8%	3	10.7%
Stomach	4	23.5%	1	5.9%	11	12.5%	3	10.7%
NPC	1	5.9%	1	5.9%	3	3.4%	3	10.7%
Pancreatic	3	17.6%	2	11.8%	5	5.7%	1	3.6%
BRCA related	13	76.5%	12	70.6%	60	68.2%	18	64.3%
